# Infective endocarditis in octogenarians. A retrospective study in a single, high-volume surgical centre

**DOI:** 10.1186/s12877-023-04345-8

**Published:** 2023-10-13

**Authors:** Valentina Scheggi, Silvia Menale, Barbara Tonietti, Jacopo Giovacchini, Stefano Del Pace, Nicola Zoppetti, Bruno Alterini, Pier Luigi Stefàno, Niccolò Marchionni

**Affiliations:** 1 Cardiothoracovascular Department, Azienda Ospedaliero-Universitaria Careggi, Florence, Italy; 2 Department of Experimental and Clinical Medicine, University of Florence, Florence, Italy; 3 Health Management Direction, Azienda Ospedaliero-Universitaria Careggi, Florence, Italy; 4 Institute of applied physics “Nello Carrara” (IFAC), National Research Council, Florence, Italy

**Keywords:** Elderly, Infective endocarditis, Features, Prognosis

## Abstract

**Background:**

Infective endocarditis (IE) is a severe disease associated with high morbidity and mortality. Little is known about the best management of elderly patients with IE. In these patients, surgery may be challenging. Our study aimed to describe IE’s features in octogenarians and to identify the independent predictors of mortality, focusing on the prognostic impact of disability.

**Methods:**

We retrospectively analyzed 551 consecutive patients admitted to a single surgical centre with a definite diagnosis of non-device-related infective endocarditis; of these, 97 (17.6%) were older than 80 years.

**Results:**

In patients under eighty, males were mostly involved with a sex ratio exceeding 2:1. This ratio was inverted in older people, where the female gender represented 53.6% of the total. Enterococci (29.8 vs. 17.4%, p = 0.005) were significantly more frequent than in younger people. Comorbidities were more frequent in elderly patients; consequently, EuroSCORE II was higher (median ± IQR 16.4 ± 21.1 vs. 5.0 ± 10.3, p = 0.001). In octogenarians, IE was more frequently left-sided (97.9 vs. 89.8%, p = 0.011). Octogenarians were more often excluded from surgery despite indication (23.7 vs. 8.1%, p = 0.001) and had higher three-year mortality (45.3 vs. 30.6%, p = 0.005) than younger patients. In elderly patients, age did not independently predict mortality, while exclusion from surgery and a high grade of disability did.

**Conclusions:**

Octogenarians with IE have specific clinical and microbiological characteristics. Older patients are more often excluded from surgery, and the overall prognosis is poor. Age per se should not be a reason to deny surgery, while disability predicts futility.

## Background

Infective endocarditis (IE) is a severe disease associated with high morbidity and mortality. The EURO-ENDO registry reported a mean age of 59.25 ± 18.03 years, with 12.0% of patients over 80 years [1]. Little is known about the best management of older patients with IE. Since surgery in advanced age patients is potentially challenging because of the high risk, the proportion of octogenarians excluded from surgery despite an indication is higher than in younger people [2]. Not surprisingly, exclusion from surgery is an independent predictor of mortality [3, 4]. Therefore, both the indication for surgery and the exclusion from it must rely on a balance of solid clinical criteria. Our study aimed to describe the IE’s specific features in octogenarians and to identify the independent predictors of mortality in this specific population, focusing on the prognostic impact of disability on admission.

## Methods

### Patient selection

We analyzed the data of 551 incident cases of non-device-related IE admitted to our department from January 2013 to November 2021. Device-related IE was defined as an infection on cardiac devices other than valve prosthesis. Data for analysis were retrieved from electronic hospital charts and were fully anonymized. The local Ethics Committee approved the study and, in accordance with Italian laws for observational studies, granted a waiver of informed consent from study participants. For all patients, we collected the following data: sex; age; the presumable entry site of the infection; history of alcohol or intravenous drug abuse; history of hypertension, diabetes, cancer, chronic renal failure (eGFR < 60 mL/min/1.73m^2^ by Cockroft-Gault formula), previous IE (first episode or relapse); white blood cells count, serum creatinine; size of IE vegetation, site of infection (mitral or aortic), type of valve (native or prosthetic), left ventricular ejection fraction, paravalvular extension, degree of valvular dysfunction (mild, moderate, severe); embolic events detected on admission; causative pathogen (determined by blood cultures, serology testing, valve culture, or polymerase chain reaction on a valve specimen, according to guidelines [5]). Transthoracic and transesophageal echocardiography were performed in all patients by experienced physicians during the acute phase of IE and the data from the first echocardiographic study were collected. Echocardiographic data included the presence, localization, and maximal length of vegetations. We followed the current international IE guidelines for diagnostic work-up and treatment strategies [5]. Surgery was defined as early when performed within 14 days [5]. Moreover, the functional status on admission was routinely assessed, by interviewing the patient and relatives, with the Barthel Index (BI), in which lower values correspond to poorer functional status and to poorer prognosis in the general population [6]. Briefly, the BI summarizes functional independence in feeding, bathing, grooming, dressing, bowels, bladder, toilet use, transfers, mobility, and stairs climbing. We categorized the BI into three groups, indicating a preserved or only mildly deteriorated overall functional independence (≥ 65/100), as opposed to a moderate (40–64/100) or a severe (≤ 39/100) disability.

### Follow-up

We calculated the follow-up duration from the time of IE diagnosis. A structured phone interview updated the follow-up of all patients to March 2022.

### Study endpoint

Identification of predictors of mortality in octogenarian patients affected by IE was the primary study endpoint.

### Statistical analysis

We used the chi-square and the Mann-Whitney or Kruskal-Wallis tests to compare respectively proportions and continuous variables with normal or non-normal distribution. We performed univariable and multivariable analyses using logistic regression and general linear models. We used the Kaplan-Meier method to estimate the univariate survival analysis and the Cox regression to identify the multivariable associations with mortality and estimate their hazard ratio with a 95% confidence interval. Multivariable analysis of all-cause in-hospital and long-term mortality was adjusted for the treatment received: surgery, medical therapy for the absence of surgical indication or exclusion from surgery for prohibitive clinical conditions. Multivariable Cox proportional hazards model included all covariates that resulted significantly different between the two groups described in Table 1 (patients under and over the age of 80) at univariable analysis and presented no missing values (age, gender, history of drug abuse, the microbiologic agent involved, left ventricular ejection fraction, type of valve affected, renal failure, hypertension, diabetes, the presence of a pacemaker, EuroSCORE II, and the BI class). We also included double valve infection and cerebral embolism since they are known prognostic factors from the literature [5]. In the cohort of octogenarian patients undergoing surgery, we repeated the multivariable Cox analysis, including the timing of surgery (early vs. delayed surgery), beyond the previous variables (Table 2). We used the Area Under the Curve (AUC) derived from the Receiver Operating Characteristic (ROC) analysis to establish the accuracy of the Cox-derived models.

**Table 1:** Demographic, clinical, echocardiographic and microbiologic characteristics of the study population, by age under or over 80 years

		Age (years)		
		< 80 (N = 454)	≥ 80 (N = 97)	p-value
Age (years, median ± IQR)		65 ± 21	82 ± 4	0.001
Female gender (N, %)		138 (30.3%)	52 (53.6%)	0.001
BMI (median ± IQR)		24.2 ± 5.0	24.2 ± 4.5	NS
Diabetes (N, %)		84 (18.5%)	20 (20.6%)	NS
Dyslipidemia (N, %)		113 (24.8%)	40 (41.2%)	0.003
Hypertension (N, %)		247 (54.4%)	76 (78.3%)	0.001
Chronic kidney disease (N, %)		98 (21.5%)	34 (35.0%)	0.005
	mild	37 (8.1%)	10 (10.3%)	0.002
	moderate	32 (7.0%)	18 (18.5%)	
	severe	13 (2.8%)	5 (5.1%)	
	dyalisis	16 (3.5%)	1 (1.0%)	
Cancer (N, %)		90 (19.8%)	27 (27.8%)	NS
Pacemaker (N, %)		47 (10.3%)	20 (20.6%)	0.005
Barthel Index (BI) class (N, %)				
	mild (BI ≥ 65/100)	310 (68.2%)	49 (50.5%)	0.002
	moderate (BI 40–64/100)	67 (14.7%)	27 (27.8%)	
	severe (BI ≤ 39/100)	77 (16.9%)	21 (21.6%)	
Drug abuse (N, %)		60 (13.2%)	0 (0%)	0.001
Vegetation length (mm, median ± IQR)		10.0 ± 11.0	9.0 ± 14.0	NS
Left-sided IE (N, %)		408 (89.8%)	95 (97.9%)	0.011
Prosthetic valve (N, %)		180 (39.6%)	51 (52.5%)	0.02
Double valve infection (N, %)		78 (17.1%)	14 (14.4%)	NS
Severe valvular dysfunction (N, %)		214 (47.1%)	46 (47.4%)	NS
Paravalvular extension (N, %)		92 (20.2%)	24 (24.7%)	NS
LVEF (%, median ± IQR)		60.0 ± 11.0	57.0 ± 11.5	NS
TAPSE (mm, mean ± SD)		21.0 ± 4.7	19.2 ± 4.3	0.025
EuroSCORE II (median ± IQR)		5.0 ± 10.3	16.4 ± 21.1	0.001
Embolism (N, %)		197 (43.3%)	33 (34.0%)	NS
Germ (N, %)				
	*Streptococci*	111 (24.4%)	21 (21.6%)	NS
	*Streptococcus bovis*	34 (7.4%)	7 (7.2%)	NS
	*Staphylococcus aureus*	92 (20.2%)	12 (12.3%)	NS
	*Coagulase negative staphylococci*	57 (12.5%)	17 (17.5%)	NS
	*Enterococci*	79 (17.4%)	29 (29.8%)	0.005
	Negative coltures	88 (19.3%)	15 (15.4%)	NS
Treatment (N, %)				
	Surgery	362 (79.7%)	69 (71.1%)	0.001
	Absence of surgical indication	55 (12.1%)	5 (5.1%)	
	Excluded from surgery	37 (8.1%)	23 (23.7%)	

BMI: body mass index. LVEF: left ventricular ejection fraction. TAPSE: tricuspid annular plane excursion

**Table 2:** Multivariable analysis of predictors of all-cause mortality in patients 80 + years of age

	HR (95% CI)	p value
Overall cohort (N = 97)		
Exclusion from surgery	9.5 (1.1–80.1)	0.038
Double valve infection	3.1 (1.4–6.9)	0.005
Barthel index < 40/100	2.7 (1.4–5.2)	0.002
Chronic kidney disease	2.3 (1.2–4.2)	0.008
Patients treated surgically (N = 69)		
Age	1.3 (1.1–1.6)*	< 0.001
Enterococci	2.4 (1.0-5.6)	0.039
Streptococci	0.2 (0.05–0.8)	0.023
Early surgery	0.3 (0.2–0.9)	0.022
Barthel index < 40/100	4.0 (1.7–10)	0.002

Chronic kidney disease: eGFR < 60 ml/min/m^2^

*HR per year

All tests were 2-sided, and statistical significance was defined as a p-value < 0.05. We performed the analyses with SPSS 23.0 and R 3.6.3.

## Results

### Patient characteristics

Patients 80 + years of age represented 17.6% of the whole population (N = 97/551), which had a median follow-up of 3.4 years (95%CI 3.2–3.6). The main demographic, clinical, echocardiographic and microbiologic characteristics of patients under and over the age of 80 are reported in Table 1.

Older patients were more frequently females and more frequently affected by dyslipidemia, arterial hypertension and chronic kidney disease (CKD). They also more frequently had a permanent pacemaker. Functional limitations were more prevalent with ageing, as indicated by a BI moderate-to-severe recorded in 49.5% of octogenarians and in 31.7% of individuals in the younger cohort. No older patient had a history of drug abuse, and, accordingly, right-sided IE was less prevalent in this group, while a prosthetic valve infection was more frequent. IE on previous TAVI (trans aortic valve implantation) was present in 2 (1%) patients under and in 3 (6%) over 80 years (p = 0.035). In keeping with older age and greater burden of comorbidities (in particular, CKD), EuroSCORE II was higher in the octogenarians’ group. Germs isolated from blood cultures were similar in the two age groups with the only exception of Enterococci, which were significantly more frequent in the older cohort. The severity of valve dysfunction, paravalvular extension, vegetation length, and left ventricular ejection fraction were similar in the two groups, whereas tricuspid annular plane systolic excursion was lower in older patients.

### Surgical treatment and mortality

Cardiac surgery was accomplished in 431/551 (78%) patients, whereas 60 (11%) were treated medically because the indication for surgery was not confirmed, and 60 (11%) were excluded from surgery, despite a surgical indication, because of prohibitive general conditions.

Octogenarians were more often excluded from surgery despite indication (23.7 vs. 8.1%, p = 0.001) and had higher 3-year mortality (45.3 vs. 30.6%, p = 0.005). When eligible for surgery, patients under and over 80 years were operated on within two weeks of IE diagnosis and underwent valvular repair in similar proportions (79 vs. 71% and 20 vs. 17%, respectively).

**Fig. 1:**
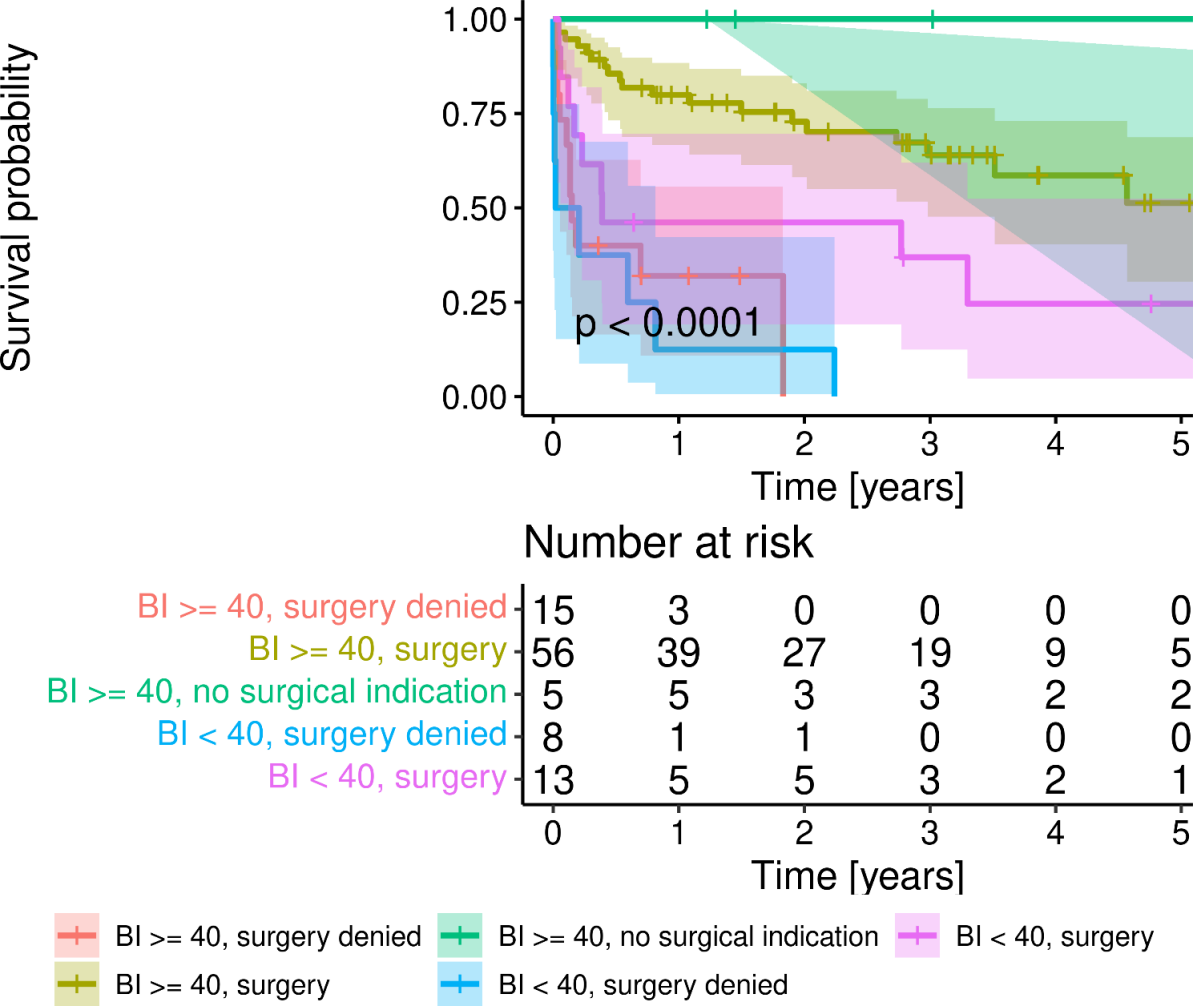
Kaplan-Meier analysis of survival probability of 97 octogenarian patients with infective endocarditis divided for Barthel index (BI) < or ≥ 40 and therapeutic strategy (surgery denied, surgery, no surgical indication)

In the older group, neither age nor EuroSCORE II were independently associated with mortality, which was predicted by CKD, presence of double valve infection, exclusion from surgery because of prohibitive clinical conditions, and pre-morbid overall functional limitations, as indicated by a BI lower than 40/100 (Table 2). Indeed, as shown in Fig. 1, patients with a pre-morbid disability, as indicated by a BI < 40, who were operated on had a poorer prognosis after surgery than those operated on who had a reasonably preserved functional (BI ≥ 40), suggesting that a limited functional capacity anticipates substantial futility of surgery even when indicated. The Barthel Index confirmed to be an independent predictor of mortality even in the subset of octogenarian patients treated surgically, as shown in Table 2. The accuracy of both models described in Table 2 was comparable with that of EuroSCORE II, with an AUC derived from ROC curves of 0.59 and 0.60, respectively.

## Discussion

The main findings of our study include the peculiar clinical characteristics of IE in octogenarians, their different therapeutic approach compared to younger patients, and the identification of pre-morbid overall functional status as a key element to be taken into account in the pre-operative assessment.

The female gender is prevalent in the older as opposed to the younger population of our study. This gender difference might rely on a protective effect of oestrogens on endothelial integrity, such as that reported to explain the delayed onset of atherosclerosis in females. As expected, older females also have a greater prevalence of comorbid conditions [7], such as CKD.

In keeping with other studies [8], the larger prevalence of enterococcal infection in our older group reinforces the importance of an accurate empiric antibiotic selection beyond the increasing frequency of nosocomial enterococcal infections and of antimicrobial resistance [9].

The length of infective vegetations was similar in octogenarians and younger patients of our series. This contrasts with the hypothesis that a delayed diagnosis of IE in older persons is the consequence of smaller vegetations [10]. However, the diagnosis of IE in the elderly always represents a major challenge because of its subtle clinical presentation [11].

As expected, octogenarians more frequently had a prosthetic valve infection, a greater burden of comorbidities and, consequently, a higher surgical risk profile as indicated by a much higher EuroSCORE II. As reported in other studies, this complexity translates into lower access to surgery and higher mortality [2]. No international guideline addresses the management of IE specifically tailored to the elderly. Notably, no previous studies have evaluated the role of disability in the pre-operative evaluation of elderly patients with IE, although other authors found age itself is not a good surrogate marker to define prognosis [2, 11].

According to our results, a Barthel Index indicating some degree of disability in performing basic everyday tasks or more complex tasks needed for independent living [12] was an independent predictor of mortality. In fact, overall functional status, as assessed by the BI, stratified the prognosis of octogenarians with IE better than EuroSCORE II, which was not independently associated with mortality. Therefore, we believe that denying surgery to older patients with IE solely based on age or on EuroSCORE II may deny them a potentially life-saving procedure; moreover, information on functional should be systematically collected in order to identify those patients in whom surgery might reveal futile.

Our study has some limitations: first, its retrospective and monocentric nature; second, changes in the clinical management of IE may have occurred over the long study period. Finally, our study has a clear referral bias of a high-volume surgical centre; therefore, the percentage of patients with a surgical indication is disproportionately higher than that commonly reported in larger, multicentre studies [1].

## Conclusions

Octogenarians represent a considerable proportion of cases of IE, with specific clinical and microbiological characteristics. Beyond the consideration of clinical elements described by guidelines, we recommend that the decision-making process in older IE patients should take into maximal account the definition of individual functional profile to improve the appropriateness of indication to surgery.

## Data Availability

The datasets used and/or analysed during the current study are available from the corresponding author on reasonable request.

## Declarations

The need for informed consent was waived by the ethics Committee Comitato Etico Regionale per la Sperimentazione Clinica della Regione Toscana in keeping with statements by the Italian Regulatory Authorities for retrospective studies:

(https://www.garanteprivacy.it/web/guest/home/docweb/-/docweb-display/docweb/5805552).

As reported in the methods section, all methods were carried out in accordance with relevant guidelines and regulations.

## Abbreviations

BI: Barthel Index
BMI: body mass index
CKD: chronic kidney disease
eGFR: estimated glomerular filtration rate
IE: infective endocarditis
LVEF: left ventricular ejection fraction
SD: standard deviation
TAPSE: tricuspid annular plane excursion
